# Telling functional networks apart using ranked network features stability

**DOI:** 10.1038/s41598-022-06497-w

**Published:** 2022-02-15

**Authors:** Massimiliano Zanin, Bahar Güntekin, Tuba Aktürk, Ebru Yıldırım, Görsev Yener, Ilayda Kiyi, Duygu Hünerli-Gündüz, Henrique Sequeira, David Papo

**Affiliations:** 1 Instituto de Física Interdisciplinar y Sistemas Complejos IFISC (CSIC-UIB), Campus UIB, 07122 Palma de Mallorca, Spain; 2 Department of Biophysics, School of Medicine, Istanbul Medipol University, Istanbul, Turkey; 3 Health Sciences and Technology Research Institute (SABITA), Istanbul Medipol University, Istanbul, Turkey; 4 Program of Electroneurophysiology, Vocational School, Istanbul Medipol University, Istanbul, Turkey; 5 Department of Neurosciences, Health Sciences Institute, Dokuz Eylül University, Izmir, Turkey; 6 School of Medicine, Izmir University of Economics, Izmir, Turkey; 7 University of Lille, CNRS, UMR 9193-SCALab-Sciences Cognitives et Sciences Affectives, 59000 Lille, France; 8 Department of Neuroscience and Rehabilitation, Section of Physiology, University of Ferrara, Ferrara, Italy; 9 Fondazione Istituto Italiano di Tecnologia, Ferrara, Italy

**Keywords:** Network models, Alzheimer's disease, Parkinson's disease, Complex networks

## Abstract

Over the past few years, it has become standard to describe brain anatomical and functional organisation in terms of complex networks, wherein single brain regions or modules and their connections are respectively identified with network nodes and the links connecting them. Often, the goal of a given study is not that of modelling brain activity but, more basically, to discriminate between experimental conditions or populations, thus to find a way to compute differences between them. This in turn involves two important aspects: defining discriminative features and quantifying differences between them. Here we show that the ranked dynamical stability of network features, from links or nodes to higher-level network properties, discriminates well between healthy brain activity and various pathological conditions. These easily computable properties, which constitute local but topographically aspecific aspects of brain activity, greatly simplify inter-network comparisons and spare the need for network pruning. Our results are discussed in terms of microstate stability. Some implications for functional brain activity are discussed.

## Introduction

Characterising the structure of brain dynamics and understanding how the demands of physiological or cognitive tasks act on it to give rise to function represent fundamental endeavours in neuroscience. Both at rest and during the execution of cognitive tasks, brain dynamics has been shown to present rich non-random spatio-temporal structure^1–7^. On the one hand, the need to respond in a fast and reliable way to changes in the environment favoured the emergence of functionally specialised segregated modules, e.g., sensory systems. On the other hand, carrying out complex tasks may sometimes involve computational sophistication exceeding single module capacity, and require interactions among them^8^. Overall, the global organisation of healthy brain activity can be thought of as a balance between these two modes^9–11^, and imbalance between segregation and integration has been associated with various pathological conditions, e.g., autism or schizophrenia^10–12^, epilepsy^13,14^, and LSD consumption^15^.

This organisation is naturally described in terms of complex networks^16,17^, wherein single brain regions or modules and their connections are respectively identified with network nodes and the links connecting them^18^. Simple functions of the connectivity contain information on the organisation at all scales of the underlying system and can therefore be used to model relations within and between brain modules, for a given coarse-graining level of that system, both in healthy brains and in neurological and psychiatric pathologies^19^. Most importantly, the brain is organised around an integration-segregation trade-off, reflecting a balance between topographically local specificity and global activity. On the other hand, the standard statistical mechanics approach to complex networks neglects, at least prima facie, the lack of translational invariance of functional brain activity in the anatomical space. Nodes and links have no topographically-specific properties, and the network is described in terms of statistical properties of the interactions^20^; to illustrate, nodes may have specific topological properties, but the mapping with the corresponding brain regions is lost in such abstract representation.

Often, studies may not set out to model brain activity but simply aim to discriminate between experimental conditions or populations. One fundamental question is then to find a way to compute differences or, somehow equivalently, distances between experimental conditions or populations. This in turn involves two important aspects: defining discriminative features and quantifying differences between them.

In the network neuroscience approach, the former aspect constitutes a particularly arduous task, due on the one hand to computational issues induced by network size and the consequent need to reduce the associated problem’s dimensionality and, on the other hand, to the multiplicity of network properties. The two main somehow interrelated questions in such an approach are then in general: what aspects of the network structure are sufficient and efficient in such a discrimination task? How can the network’s dimensionality be reduced without affecting its discriminatory power? On one hand, which features possess discriminatory power may be difficult to predict. Often, the first considered feature dimension is related to the system’s representation in the anatomical space. While the integration–segregation literature suggests the discriminating feature to be a combination of local topographically-specific and distributed and network-like, a recent study^21^ pointed to the possibility that experimental conditions may be identifiable by their relations across the whole network in a way that is at least partially independent of topographical localisation. This study showed that the hierarchy of dynamic connectivity strengths can discriminate between different patient populations. It also showed that the dynamics of connectivity may be more important than its spatial fine-graining in discriminating between conditions; and that variability has a prominent role in healthy biological systems and in pathology. On the other hand, computational costs and graphical representability often force reducing the analysis to the subnetwork created by the strongest links, or by links selected according to other criteria^22^. However, weak links have been shown to have a strong impact on network topology^23,24^, dynamics and on the processes taking place on it^25,26^. Importantly, weak links may be key to brain function^27^, but also to the discrimination between brain pathologies^21^, and retaining higher percentages of links may improve classification accuracy^28^.

Quantifying differences in connectivity and in network properties across conditions or populations also constitutes an arduous task. Functional networks typically differ in size^29^, and efforts to tackle this difficulty may distort the functional meaning of the reconstructed network. Moreover, choosing the appropriate distance may also be non-trivial^30^. Finally, the multiplicity of metrics is inevitably associated with multiple comparison risks.

Here we propose to discriminate networks of brain activity using the stability of some property of the dynamical network. In the simplest case, this involves discriminating populations by the stability of the strongest link in the network. This principle is naturally extended to the stability of higher-order structures in the network induced by dynamic connectivity. Importantly, these properties constitute in general local but topographically aspecific features. In this sense they would provide complementary information with respect to both topographically-specific or global aspects of brain activity of existing analyses, and would provide a simple criterion greatly simplifying both feature choice and quantification of differences across experimental conditions. Ranked link stability induces two variables (network feature rank stability and its associated time scale). Stability can in principle be measured at all scales from microscopic (links, nodes) to mesoscopic and macroscopic scales. Here, we quantify link, node centrality and clustering coefficient stability associated with the resting brain activity of various neurological populations: amnesic mild cognitive impairment (MCI), Alzheimer’s disease (AD), Parkinson’s disease with MCI (PD-MCI) and Parkinson’s disease dementia (PD-D). We further validate our results using an auxiliary data set comprising Parkinson’s disease patients and matched control subjects.

## Results

### Stability of network features

For each subject in a given group, and for each available $\left\lfloor l/w \right\rfloor$ functional networks (where *l* is the total length of the available time series, and *w* the size of the considered non-overlapping windows, see “Materials and methods” for details on the reconstruction process), we extract the link with the largest weight. Note that this here corresponds with the link of highest absolute linear correlation value, but any other correlation or causality metric could be used. For each subject, the link that most frequently turns out to be the strongest is then selected; and the corresponding probability is calculated according to a binomial distribution $\pi = B(n, p, k)$, where:

- *n* is the number of trials, here the number of windows for a subject, i.e. $\left\lfloor l/w \right\rfloor$.
- *p* the success probability of each trial, or the probability of finding the target link as the strongest one. The probability is thus the inverse of the number of possible links, i.e. $1 / (N (N-1) / 2)$.
- *k* is the number of successful trials, i.e. the number of times the most frequent strongest link has appeared.

$\pi$ thus represents the probability of obtaining the most frequent strongest link at random, i.e. if networks were completely independent; and can thus be seen as the *p* value, with the null hypothesis being the independence of consecutive networks. Here the value $\ln \pi$ is represented. Insofar as obtaining very stable strongest links is highly improbable, $\ln \pi \ll 0$ suggests that a subject’s most frequent strongest link is stable across time; in other words, $\ln \pi$ is inversely proportional to the stability of such link. Similarly, the window size *w* for which $\ln \pi$ is minimised indicates the time scale at which such stability is mostly observed. A similar analysis has also been carried out for the stability of the node with the highest strength centrality in each network, i.e. the node with the largest sum of weights of links connected to it; and for the stability of the node with the largest (local) clustering coefficient^31^.

### Stability of the strongest link

**Figure 1:**
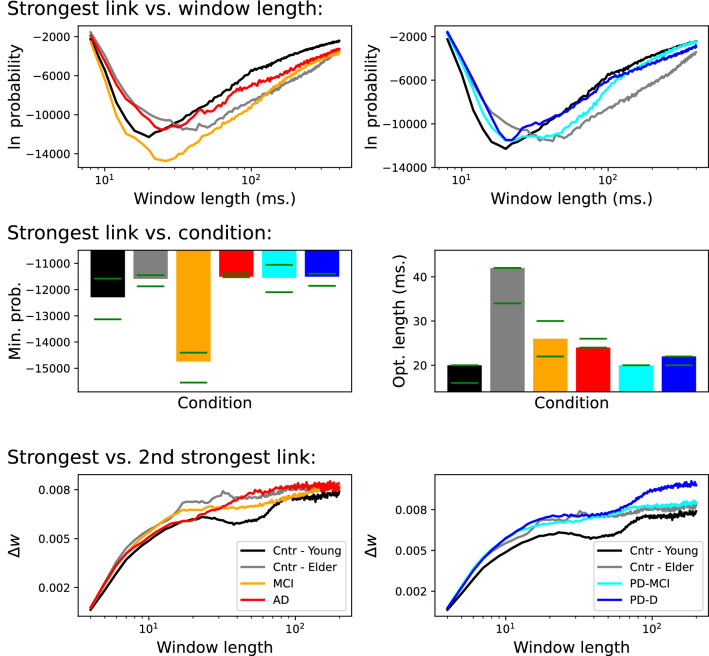
Stability of the strongest links. (Top panels) Evolution of the logarithm of the probability of finding the most frequent strongest link, as a function of the length of the window used to reconstruct the networks, for the six considered groups. (Centre panels) Peak value of the top graphs, both in terms of the minimal probability logarithm (left panel) and of the optimal window length yielding that probability (right panel). Green lines indicate the maximum and minimum obtained when deleting one subject. (Bottom panels) Average distance between the strongest and the second strongest link. In the top and bottom panels, patient groups are distributed in two columns for the sake of clarity.

We applied the previously described methodology to detect how stable the strongest link is for each subject. Specifically, the top two panels of Fig. 1 report the evolution of $\ln \pi$ as a function of the window length *w*, averaged over all patients belonging to the six groups here considered (see Materials and Methods for details on the data sets). Small window lengths are associated with unstable strongest links; this is to be expected, as windows of size $\approx 10$ ms can only detect high-frequency (and potentially noisy) trends. The same result is predictably found in the other extremum, reflecting the intrinsically fluctuating nature of resting brain activity.

Strong minima can be found for window lengths between 20 and 40 ms, as synthesised by the two central panels of Fig. 1. The optimal *w* is smaller for young control subjects, but strongly increases for elder controls. For patients, the optimal *w* is shorter than the one associated with elderly control subjects, though still slower than the one characterising young control subjects. Regarding the magnitude of the minimum probability, this is especially small for MCI patients. While this probability has a physical interpretation (as the probability of finding a strongest link in random networks as stable as what observed, and hence is inversely proportional to the stability of such link), it can also be interpreted as a topological feature of the functional network itself, and as such can be used to assess differences between groups—as demonstrated below. Horizontal green lines in these two panels further report the maximum and minimum of both metrics, when one of the subjects belonging to that group is deleted—hence providing an estimation of the sensitivity of the metrics, and of the statistical significance of results.

Figure 1’s bottom panels report the evolution of the average distance between the strongest and the second strongest link in the ranking, as a function of the window size. Such distance is calculated as $\Delta w = \log _2 s_{1} / s_{2}$, where $s_i$ is the strength of the *i*-th link in the ranking. Distances appear to be very small for all groups, with minimal differences for control subjects in the 40–150 ms range. Note that the minimum observable in the top panels is not present. Thus, it appears that the stability of the most important link is not a direct consequence of varying distance to the second most stable one.

### Stability of multi-link structures

**Figure 2:**
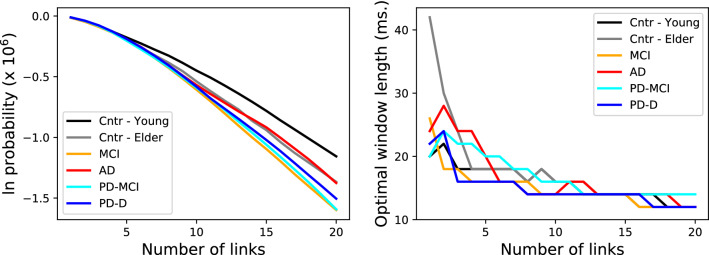
Stability of multi-link structures. (Left) Evolution of the logarithm of the probability of finding a consistent set of strongest links, as a function of the number of links, and for the six groups here considered. (Right) Evolution of the window length yielding the maximal stability, as a function of the number of links considered.

The two panels of Fig. 2 respectively depict the evolution of the probability, and of the optimal window length, when multiple strongest links are considered at the same time, as a function of the number of such links. It can be appreciated that larger structures are stable only for small values of *w*. According to the previous hypothesis, links calculated with small *w*s are influenced by noise. Nevertheless, here we are now considering a larger number of strong links at the same time; noise may then change their relative ranking, but not their belonging to the top set. In synthesis, larger structures are more stable when considering shorter time windows.

### Stability of node-based topological features

**Figure 3:**
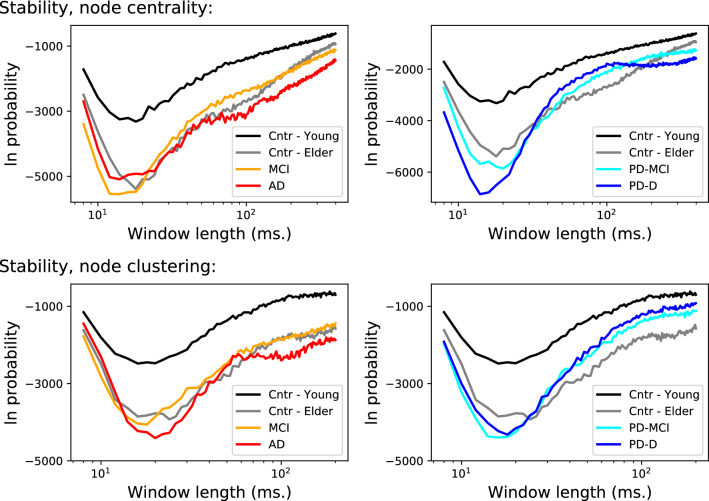
Stability of node-based features. (Top panels) Evolution of the logarithm of the probability of finding the most frequent most central node, as a function of the length of the window used to reconstruct the networks, and for the six groups here considered. (Bottom panels) Evolution of the logarithm of the probability of finding the most frequent node with highest clustering coefficient. Patient groups are distributed in two columns for the sake of clarity.

The top and bottom panels of Fig. 3 represent the stability of the node with respectively the highest centrality and clustering coefficient. As expected from the results of Fig. 2, the optimal window length is in general smaller than 20 ms, as we are here analysing structures larger than a single link. In both cases, the most important node is less stable in young control subjects, both elderly controls and patients having a much lower $\pi$.

### Intra-group stability

**Figure 4:**
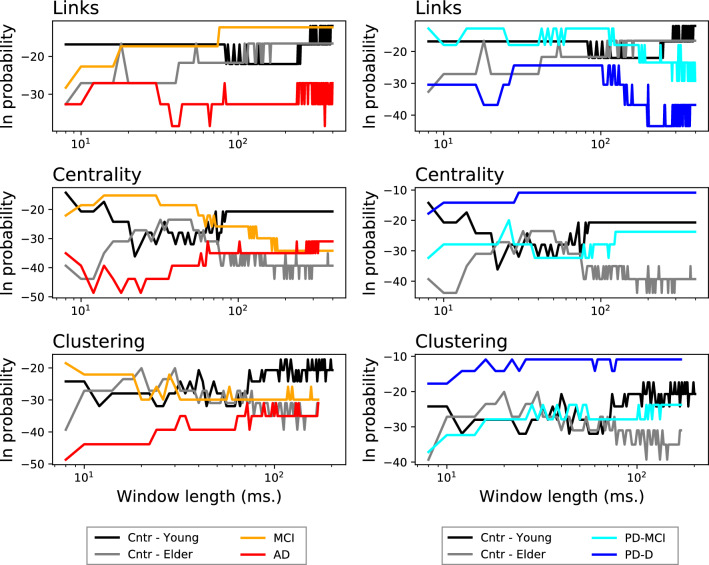
Intra-group stability. Evolution of the logarithm of the probability of finding the most frequent link with highest weight (top panels), and of the most frequent node with highest centrality (central panels) and highest clustering coefficient (bottom panels), as a function of the length of the window used to reconstruct the networks. Colour code of groups as in previous figures.

We further analyse how the strongest links, and the nodes of highest centrality and clustering, are stable not only within each subject, but also across subjects belonging to each group. This is achieved by firstly splitting the available time series into non-overlapping windows of size *w*; reconstructing the individual functional networks; and identifying the link or node of maximal topological feature—that is, as done when analysing one single subject. Secondly, results are aggregated for all subjects belonging to the same group, thus yielding a single probability $\pi$ per group. Results are presented in Fig. 4; as in the previous cases, what is represented is the evolution of the logarithm of the probability of finding the same link (or node) being the strongest one (respectively, the most central one) in multiple subjects, as a function of the length of the window used to reconstruct the functional networks.

It can be appreciated that control subjects’ behaviour, both young and elderly, and of MCI patients is qualitatively similar. On the other hand, a large difference can be observed for AD and PD-D in the node-based metrics: while the former is characterised by stability across subjects, in the latter the logarithm of the probability increases up to $-10$. In other words, the most central node and the node with highest clustering frequently coincide for AD patients, but generally differ for PD patients.

### Topology and neuropsychological data

Beyond analysing how the stability of some network structure changes between different conditions, it is also of interest to see how it is related to people’s demographic and cognitive information. Towards this aim, Table 1 reports the coefficients of the Spearman’s rank-order correlation between several attributes, and the stability of three network structure, both in terms of their probability of appearance and the best window length at which they appear. Correlations are generally small, as can be expected with the drastic information compression associated with ranked feature stability. It is nevertheless worth noting the positive correlation between age and best window lengths, for all three metrics, for control subjects; and how such correlation becomes mostly negative for patients. This may point towards a mechanism whereby brain dynamics becomes faster but at the expense of increased randomness. Furthermore, education level positively correlates with best window length to obtain a stable strongest link (statistically significant at $\alpha = 0.01$), but negatively with node centrality and clustering stability. Higher education seems to stabilise the strongest link, but also introduces more flexibility at the network meso-scale. Moving to the cognitive assessment of patients, only the Geriatric Depression Scale presents a statistically significant correlation, and specifically a negative relationship with the best window length for observing a stable strongest link, consistent with a general slow-down in dynamical transitions in depression^32,33^.

**Table 1:** Spearman’s rank-order correlation coefficients between topological metrics (columns) and demographic and cognitive indices (rows).

	Strongest link		Node centrality		Node clustering	
	m.p.	b.w.l.	m.p.	b.w.l.	m.p.	b.w.l.
Age (control)	0.123	0.272	0.059	0.139	0.091	0.162
Age (patients)	$-$ 0.037	$-$ 0.260	$-$ 0.144	$-$ 0.073	$-$ 0.177	$-$ 0.013
Education (control)	0.317	0.284	0.177	$-$ 0.136	0.129	$-$ 0.262
Education (patients)	0.092	$-$ 0.004	0.137	0.113	0.062	0.075
MMSE	0.011	0.070	0.086	0.015	0.084	$-$ 0.149
GDS	$-$ 0.078	$-$ 0.241	$-$ 0.057	$-$ 0.163	$-$ 0.059	$-$ 0.103
OVMPT	0.018	0.079	0.126	0.035	0.106	$-$ 0.152
Categorical fluency	$-$ 0.009	$-$ 0.023	0.095	0.093	0.063	$-$ 0.159
Phonemic fluency	0.018	0.130	0.117	0.081	0.097	$-$ 0.047
Language	0.068	$-$ 0.032	$-$ 0.087	0.059	$-$ 0.040	0.013

*m.p.* minimum probability, *b.w.l.* best window length, *MMSE* Mini-Mental State Examination, *GDS* Yesavage Geriatric Depression Scale, *OVMPT* Oktem Verbal Memory Processes Test.

In order to further assess the explanatory power of the six metrics reported in Table 1, several Ordinary Least Square (OLS) linear regressions have been fitted, in which the six metrics (i.e. the topology of the network) are taken as explanatory variables, and demographic and cognitive indices as the response variables. Finally, Table 2 reports the coefficient of determination $R^2$ of the fits, i.e. how much the topological properties are able to explain the observed variable values. In line with what previously seen, the $R^2$ is especially large in the case of age and education of control subjects, and still significant in the case of patients and of the Geriatric Depression Scale.

**Table 2:** Coefficient of determination $R^2$ for Ordinary Least Squares linear models between the six topological metrics included in Table 1, and the demographic and cognitive indices.

	$$R^2$$
Age (control)	0.3367
Age (patients)	0.1071
Education (control)	0.3401
Education (patients)	0.1603
MMSE	0.0279
GDS	0.1051
OVMPT	0.0337
Categorical fluency	0.0532
Phonemic fluency	0.0562
Language	0.0246

### Discrimination power

**Figure 5:**
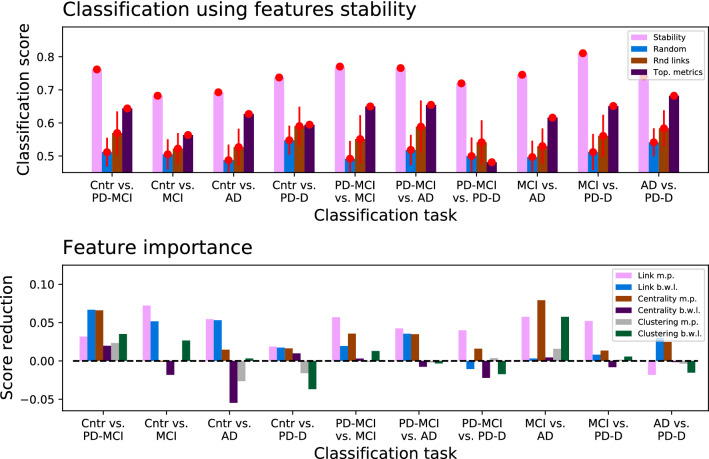
Classification using network features stability. (Top panel) Classification scores obtained by a Random Forest model, for all classification tasks (represented as groups of four bars), and using four different network features: the stability here proposed, composed of the best window length and minimum probability for the strongest link, and highest centrality and clustering nodes (pink columns); same features, but with the class of each subject randomly shuffled (blue columns); the weight of six links chosen at random (brown columns); and four standard network-wide topological metrics (purple columns). Columns represent the average over 100 random realisations, and red whiskers the corresponding standard deviation. (Bottom panel) Reduction in the classification score when one of the six network stability metrics is not used in the training.

One important aspect to be elucidated is whether the proposed stability of the topological features can be used as a way to distinguish between groups of subjects. In order to achieve this, each subject has been described by six features, i.e. the $\ln \pi$ and *w* of the strongest link and of the nodes with highest centrality and clustering. We have then executed different binary classification tasks, in which a Random Forest model^34–37^ has been trained to correctly classify subjects as belonging to a group, considering all possible pairs of conditions. The final classification scores, measured as the average accuracy over 100 independent realisations and using a Leave-One-Out cross-validation^38^, are reported in Fig. 5 (top panel, pink columns). In order to put such scores in context, the same plot also reports the classification scores in three validation scenarios: (1) by using the same features, but randomly shuffling the original class labels to obtain a random baseline; (2) using the weight of six links, chosen at random at the beginning of the classification process; and (3) the weight of two random links, plus the centrality and clustering of two random nodes. Note that, in all cases, the number of features has been kept to six, in order to provide a fair comparison and potentially the same amount of information; for this same reason, more sophisticated models using as input the whole network, as e.g. based on Deep Learning^39–42^, have not been considered. The classification score obtained with the network feature stability fluctuates between 0.7 and 0.8, and, most importantly, is substantially higher than what yielded by the other alternatives. While promising, these results have to be interpreted with due caution, due to the reduced number of subjects comprising each group.

As customary in machine learning, the previously trained models can also be used to assess the relative importance of the six stability metrics. This is done by training an independent model over the same subjects, not including the feature to be analysed; for then calculating the drop in the classification score. The larger this drop, the more important was the considered feature for achieving a correct classification, and hence the larger the quantity of information it encodes. Results are reported in the bottom panel of Fig. 5. While the importance of each feature varies depending on the pair of conditions considered, the $\ln \pi$ and *w* of the strongest link, and in less the degree the $\ln \pi$ of the node with highest clustering, seem to the best most relevant ones.

### Validation and generalisability

As a final point, we assess the validity and generalisability of the results here presented. While the main data set comprises a large number of subjects (98 in total), the fact that they are divided in six groups reduces the statistical significance of any test between them. For instance, a Kolmogorov–Smirnov test between the optimal window length for detecting the strongest link (see Fig. 1) only yields *p* values below 0.01 for the pairs control elder—control young and control elder—PD-MCI, due to the low number of samples in each group. In order to validate these results beyond the intervals reported in the central panels of Fig. 1, and the classification task above described, we here consider two different strategies: a data set upsampling, and the use of a complementary data set.

**Figure 6:**
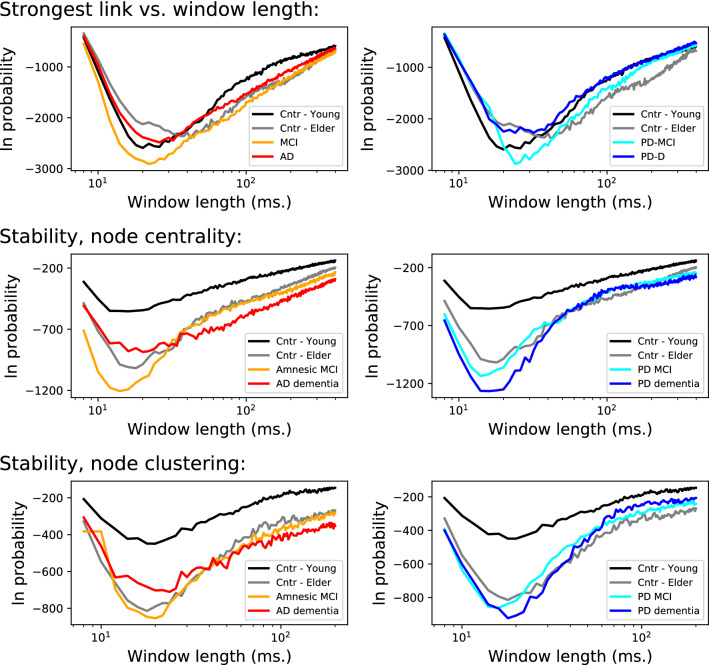
Results for the upsampled data set. Evolution of the logarithm of the probability of finding the most frequent link with highest weight (top panels), and of the most frequent node with highest centrality (central panels) and highest clustering coefficient (bottom panels), as a function of the length of the window used to reconstruct the networks. Metrics are here calculated by dividing each subject time series in five parts, and considering them as separate subjects. Colour code of groups as in previous figures.

In the first case, we split each subject’s time series in five equal parts, and then considered each part as an independent subject. This not only increments the number of (virtual) subjects in each group, but also allows to test whether the results are valid even when shorter time series are available; or, in other words, if previously shown results are stable over time. Figure 6 reports the results of this analysis, and specifically the evolution of the stability of the strongest link (top panels), most central node (central panels) and highest clustering node (bottom panels). It can be appreciated that curves are very similar to those of Figs. 1 and 3, with the minor exception of the node centrality and clustering of Amnesic MCI patients, which is slightly larger (i.e. the probability is lower) than for the original data set.

**Figure 7:**
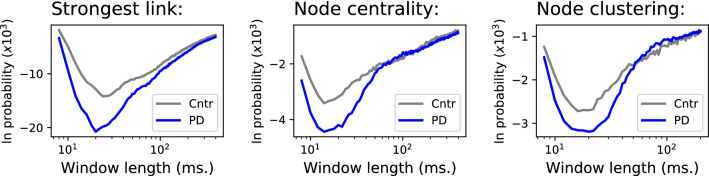
Evolution of the stability of the strongest link (left panel), most central node (central panel) and node with higher clustering (right panel) in the PD validation data set.

As an additional test for the generalisability our approach, we performed the same analysis on an independent data set of EEG recordings, including PD patients and matched control subjects (see “Materials and methods” for details on the data sets). Figure 7 reports the evolution of the stability of the strongest link, and of the most central and highest clustering nodes. Results are once again comparable with Figs. 1 and 3, with the exception of the stability of the strongest link, which is much higher than in the main data set. It is nevertheless worth noting that any comparison must be taken with caution, as for instance ages are not exactly matched between groups, e.g. control subjects are older and PD patients are younger in the validation data set.

## Discussion and conclusions

Complex networks constitute a straightforward and versatile representations of brain activity. However, comparing networks is in general an arduous tasks. Here we propose a method that greatly simplifies it, wherein networks are characterised in terms of the temporal rank stability of link and node-based features. We used ranked stability to compare resting brain activity of patients suffering from various diseases with healthy young and ageing subjects.

Our results show that link stability probability is affected in some pathologies, but not in others. Likewise, node centrality appears to be less stable for young than for elderly control subjects and for subjects with pathologies. The time scale at which link stability peaks turned out to maximally differ between young and elderly control subjects, whereas for all the considered pathologies, the optimal window length appeared to fall in between that of these two groups.

Normal ageing was characterised by decreased link stability with respect to young control subjects, but also most remarkably by a corresponding widened optimal window length, both for single link rank and for at least the most strongly connected links. On the other hand, node centrality and clustering stability turned out to be more persistent than for young healthy controls. Overall, this pattern is consistent with a picture wherein essentially normal function is maintained by compensating the slight decrease in connectivity and increased hub persistence by an overall slower dynamics, and with the idea that a certain level of flexibility is the hallmark of healthy biological systems^43^.

As in healthy ageing, all pathologies considered in this study, with the notable exception of amnesic MCI, were associated with more unstable strongest links with respect to young control subjects, but optimal window length appeared to be considerably shorter than that of normal ageing, though still longer than those of the young control group. Interestingly, a recent electrophysiological study showed that the prodromal stages of AD dementia are characterised by cortical hyperconnectivity, particularly in posterior regions, and that hyperconnectivity disappears at later stages of the disease, suggesting that it is an early electrophysiological feature of dementia^44^. Our results would in turn suggest that in addition to the total amount of connectivity^45–47^, the stability of a small subset of connections at various scales might also be used for this purpose. A noteworthy aspect is represented by the differential effect of neurodegeneration in AD and PD. The trajectory towards AD is characterised first by increased ranking stability and decreased optimal window length with respect to normal ageing, at the MCI stage, then by a reduction in fully-fledged AD. However, the pattern for node centrality and clustering ranking stability was indistinguishable from what the one observed for normal ageing. Conversely, for PD patients, MCI subjects were not associated with more stable link ranking, but only with considerably shorter optimal time windows with respect to healthy ageing. On the other hand, node centrality ranking appeared to be increasingly stable from MCI to fully-fledged PD dementia, though over the same optimal time-window as in normal ageing.

For control subjects, age and, to some extent, education level affected the optimal window length, rendering links more persistent. On the other hand, for pathological states, the optimal window length was negatively correlated with age and depression scores, but was not affected by education level. Finally, depression severity appeared to shorten the optimal time window length at all scales, a result consistent with reported alterations in functional connectivity in depression^48^.

These results can be interpreted in a straightforward way by observing that in essence what link stability documents is some property of neural microstates. Microstates are quasi-stationary segments of duration $l \le 150$ ms^49,50^ where the brain activity field remains stable, punctuated by abrupt changes to new configurations^51,52^. These stable segments were proposed to be “atoms of thought”^53^, which may correspond to different information processing steps. Shrinking microstate durations have been associated with various pathologies, including schizophrenia^54–59^, AD^60–62^, PD^63–65^, depression^32,33^, mood alterations^66^, or panic disorder^67^. Shortening of specific microstate classes, in combination with altered microstate syntax, have been interpreted to reflect disturbances in the information processing stream^49,50^. Interestingly, various studies found not only that patients suffering from AD had microstates of shorter duration compared to healthy elderly controls^68–70^, but also of altered dynamics^62^. On the other hand, microstates were found to be of abnormal duration for PD patients, some topographical maps with significantly shorter and other ones with significantly longer durations with respect to matched controls^64^. Since, control subjects for AD and PD populations are typically over 60 years of age, our results are at least partially consistent with microstate literature. Finally, depression was also found to be associated with shortening^32^ and more generally alterations of microstate morphology and dynamics^33^.

From a methodological view-point, it is important to compare ranked stability with microstate analysis. In essence, ranked link stability can be thought of as microstates’ topographically aspecific minimal core at a given spatial scale. In this vein, cluster stability represents a higher-order microstate core; conversely, standard microstaste topographical maps can be thought of as whole brain stability. In microstate analysis, brain activity is described in terms of occurrence rate, duration, and transition sequence of topographical maps. Although ranked feature stability mainly deals with the first aspect and to some extent with the second, it can in principle produce comparable analyses, albeit in an arguably more cumbersome form for the latter two. On the other hand, ranked stability presents some important advantages with respect to standard microstate analysis: (1) it minimally depends on discretionary choices such as those involved in segmentation procedures in standard microstate analysis; (2) it explicitly takes into account the relational structure of brain activity, and (3) it can potentially parse microstates’ topology at all scales, from the microscopic ones of links and nodes to the global scale of standard microstate topographic maps.

More generally, the proposed method shows that ranked network features, that are spatially local but topographically aspecific aspects of resting neural activity, together with the time scale that each of these aspects induces at a given spatial scale, contain surprisingly rich information on the underlying brain activity. In particular, information on connectivity limited to the strongest link and its persistence, irrespective of its topographical and temporal localisation, may be sufficient to discriminate resting brain activity associated with different populations. Thus, our results add an additional feature to a list of already well-documented aspects of brain activity with discriminatory power, including brain topography, connectivity and its hierarchy and topological properties. On the negative side, it should be noted that, insomuch as it requires reconstructing multiple functional networks at different temporal scales, the proposed method entails a high computational cost and can only be applied to long time series, as e.g. those recorded in a resting state.

## Materials and methods

### Main data set, control subjects and patients recruiting and selection

Ninety-eight participants were included in the study with six different groups, namely: healthy young, healthy elderly, MCI, AD, PD-MCI, and PD-D. Demographic information of the participants is presented in Table 3.

The study included participants with the diagnosis of amnestic MCI (aMCI) according to the NIA-AA criteria^71^; with PD-MCI according to the Movement Disorder Society (MDS) Level2 criteria^72^ and age-, gender- and education-matched healthy elderly controls. Patients with aMCI and PD-MCI were recruited from an outpatient memory clinic and a movement disorders outpatient clinic of a university setting, respectively. Healthy controls were recruited from various community sources.

The healthy elderly volunteers were included when no neurological abnormality or no global cognitive impairment (Mini-mental State Examination (MMSE) score $\ge 27$) were determined. Inclusion criteria for amnestic MCI were: (1) living independently in the community, (2) memory problem as defined with performances $\ge 1.5$ standard deviations below for age and education matched controls in a set of neuropsychological tests, and (3) with no impairment of daily living activities, clinical dementia rating (CDR) score of 0.5. The inclusion criteria for the young healthy controls were: no history or presence of any neurological and psychiatric abnormalities, no history of drug and alcohol abuse and/or Mini Mental State Examination (MMSE) score of $\ge 27$.

The inclusion criteria for PD-MCI patients were diagnosed according to: (1) a cognitive impairment defined by performances $\ge 1.5$ standard deviations below the normative scores in two neuropsychological tests assessing same cognitive domain or in tests evaluating two different cognitive domains; (2) stable treatment with dopaminergic medication and successful control of motor symptoms; (3) Hoehn and Yahr stage III or less; and (4) no dementia according to the Movement Disorder Society (MDS) clinical diagnostic criteria.

The exclusion criteria for all participants are as follows: (1) history of neurological and/or psychiatric including evidence of depression as demonstrated by Yesavage Geriatric Depression Scale scores higher than 13^73^; (2) presence of nonstabilized medical illnesses; (3) history of severe head injury and alcohol or drug misuse; (4) using any psychoactive drugs or cognitive enhancers including acetylcholinesterase inhibitors; (5) presence of vascular brain lesions, hydrocephalus, or a brain tumor in MRI.

The exclusion criteria for the PD-MCI patients were as follows: (1) a history of other neurological diseases; history of visual hallucinations; (2) severe tremor or dyskinesias preventing EEG recordings, and (3) treatment with subcutaneous apomorphine, jejunal levodopa, or deep brain stimulation. All participants with PD-MCI were using dopaminergic medication, including levodopa and/or dopamine agonist and/or monoamineoxidase B (MAO-B) inhibitor.

Probable PD-D diagnosis was made according to the Movement Disorder Society (MDS) Level 1 criteria^74,75^. Probable PD-D was diagnosed when all the following five criteria were satisfied: (1) Diagnosis of PD according to United Kingdom Parkinson’s Disease Society Brain Bank Criteria^76^; (2) The development of PD prior to the onset of dementia; (3) PD associated with a decreased global cognitive cognitive score which was defined as a score of $\le 24$ on the Mini Mental Status Examination (MMSE)^77^; (4) cognitive deficiency that impair daily life; (5) impairments found in more than one cognitive domain. All participants underwent complete neurological structural magnetic resonance imaging (MRI), and laboratory examinations and an extensive battery of neuropsychological tests.

All patients with Alzheimer’s disease dementia were diagnosed according to the National Institute of Aging-Alzheimer’s Association diagnostic guideline^78^. The inclusion criteria for AD patients included: (1) impairment of two or more cognitive domains; (2) impaired daily living activities with CDR score of 1 or 2. The exclusion criteria for AD patients were history or presence of any other neurological and/or psychiatric disorders including depression, traumatic brain injury, vascular brain lesions, and alcohol or drug misuse. All AD patients were on cholinesterase inhibitor drugs (donepezil; 5–10 mg per day and rivastigmine; 6–9.5 mg/24 h per day), some patients were on memantine (10–20 mg per day) in addition to cholinesterase inhibitors. All participants underwent a comprehensive neuropsychological test battery, neuroimaging, and neurological examination. Participants with depressive conditions, assessed as a Geriatric Depression Scale $> 13$, were excluded from the study^79,80^.

The study conformed to the principles of the Declaration of Helsinki. All participants and/or their relatives provided informed consent for the study, which was approved by the local ethical committee (Istanbul Medipol University Ethical Committee, Report No: 10840098-604.01.01-E.8374).

**Table 3:** Demographic data of the subject comprising the main data set.

Subject group	Size	Of which men/women	Avg. age (std.)	Avg. years of education (std.)
Control (young)	18	9/9	24.1 (3.68)	15.5 (1.54)
Control (elder)	19	11/8	69.1 (7.25)	10.9 (4.67)
MCI	16	7/9	70.4 (5.05)	9.4 (5.88)
AD	19	5/14	73.2 (5.68)	8.8 (4.40)
PD-MCI	14	9/5	71.1 (6.63)	11.4 (5.18)
PD-D	12	9/3	73.0 (6.71)	5.9 (5.21)

### Main data set, electroencephalographic data recording

EEG was recorded in two different centres (The Istanbul Medipol University Hospital, REMER, Clinical Electrophysiology, Neuroimaging, and Neuromodulation Laboratory and the Izmir Dokuz Eylül University Multidisciplinary Brain Dynamics Research Center) with the same recording system and recording protocol. EEGs were recorded in a dimly lit, soundproof, electrically shielded room. A BrainAmp 32-channel DC system (Brain Product GmbH, Germany) was used for recordings with a 500 Hz sampling rate and 0.001–250 Hz band limits. Elastic caps (EasyCap GmbH, Germany) on which 32 Ag/AgCl electrodes were placed according to the 10/20 system were used for EEG recording. All electrode impedances were kept below approximately 10 k$\Omega$. As the online reference two additionally linked electrodes (A1+A2) were placed on the earlobes. Also, two electrodes were used as the electrooculogram which was placed on the medial upper and lateral orbital rim of the left eye with Ag/AgCl electrodes.

Spontaneous EEG recordings were performed in sessions of 8 min (i.e. approximatively 240,000 data points per channel and subject), of which the first four corresponded to an “eyes open” condition, and the latter four to “eyes closed”. A black screen, specifically a 19-in. computer monitor, was presented to subjects throughout the EEG recording, without fixation cross. The experimenters watched the subjects with video monitor during the EEG recordings.

No additional data preprocessing has been carried out, and, unless otherwise specified, broadband signals have been used.

### Validation data set, participant recruiting and electroencephalographic data recording

To support the validation of results obtained in the main data set previously described, a second EEG data set of PD patients was recorded at Istanbul Medipol University Hospital in Istanbul. PD patients were diagnosed according to the criteria of “United Kingdom Parkinson’s Disease Society Brain Bank”^81^. The Unified Parkinson’s Disease Rating Scale (UPDRS) was used in order to determine the clinical features of PD; and the Hoehn-Yahr scale^82^ was used to determine the disease stage. A total of 74 patients (ages 56–86, median of 74) and 22 matched control subjects (ages 54–89, median of 67) compose this validation data set. All patients with PD were evaluated 60–90 min after their morning dose of levodopa for the EEG recordings.

The EEG signals were recorded in a dimly isolated room with a Brain Amp 32-channel DC system machine (Brain Product GmbH, Germany) from 32 different electrodes which were arranged according to the international 10/20 system. The sampling rate was 500 Hz with band limits of 0.01–250 Hz. All impedances were kept below 10 Kohm and two additional linked earlobe electrodes (A1+A2) served as reference electrodes. Electro-oculogram was recorded with two electrodes placed in the medial upper and lateral orbital rim of the left eye.

As in the case of the previous data set, spontaneous EEG recordings correspond to two 4-min sessions, respectively for “eyes open” and “eyes closed”, with a 19-in. computer monitor performing as a black screen without fixation cross. No additional data preprocessing has been carried out and broadband signals have been used.

The study conformed to the principles of the Declaration of Helsinki. All participants and/or their relatives provided informed consent for the study, which was approved by the local ethical committee (Istanbul Medipol University Ethical Committee, Report No: 10840098-51).

### Reconstructing functional networks

For each subject, the original data comprised a set of $N = 32$ time series *X*, corresponding to the 32 EEG channels, each one of length *l*. In what follows, the notation $X ^{(t)} _c$, with $t \in [1, l]$, will be used to indicate the *t*-th element of the time series *c*. These time series are split into non-overlapping windows *W* of size *w*, yielding a total of $\left\lfloor l/w \right\rfloor$ windows. In a way similar to the previous notation, $W^{(i)}_c$ denotes the *i*-th window of the time series *c*. A functional network is then created for each window, composed of 32 nodes (one for each EEG sensor). The weight of the link between nodes *j* and *k* is then given by the absolute value of the Pearson’s linear correlation between time series $W^{(i)}_j$ and $W^{(i)}_k$.

### Python software library

As a complement to this contribution, we make public a Python software library with the implementation of the methodology here proposed. In order to preserve the open nature of the methodology, the library is structured in a flexible way, in which for instance the user can provide his/her own functions for reconstructing the functional networks and calculating topological metrics. The library is freely available at https://gitlab.com/MZanin/network-feature-stability. We welcome readers to send us comments, suggestions and corrections, ideally using the “Issues” feature of GitLab.
